# A Scoping Review of the Photographic Assessment of Donor Liver Steatosis in Transplantation Using Artificial Intelligence

**DOI:** 10.1111/ctr.70433

**Published:** 2026-01-31

**Authors:** Georgios Kourounis, Samuel J. Tingle, Ali Elmahmudi, Brian Thomson, Robin Nandi, Emily Thompson, Barney Stephenson, James Hunter, Hassan Ugail, Neil S. Sheerin, Colin Wilson

**Affiliations:** ^1^ Translational and Clinical Research Institute Newcastle University Newcastle upon Tyne UK; ^2^ NIHR Blood and Transplant Research Unit at Newcastle University and Cambridge University Newcastle upon Tyne UK; ^3^ Institute of Transplantation The Freeman Hospital Newcastle upon Tyne UK; ^4^ Faculty of Engineering and Informatics Bradford University Bradford UK; ^5^ Department of Research Software Engineering Newcastle University Newcastle upon Tyne UK; ^6^ Nuffield Department of Surgical Sciences University of Oxford Oxford UK

**Keywords:** artificial intelligence, liver transplantation, machine learning, steatosis

## Abstract

**Introduction:**

Accurate evaluation of liver steatosis and overall organ quality is critical for optimizing safe organ utilization in liver transplantation. Recent advances in computer vision offer promising tools to standardize and enhance this process. This review maps the current evidence on AI‐enabled photographic evaluation of liver steatosis and identifies areas for future development.

**Methods:**

A scoping review of the literature, including searches of PubMed, SCOPUS, and Web of Science, was conducted to identify studies published from inception to 27/03/2025 reporting on the development of AI‐enabled tools for assessing liver organ quality from photographs taken during the donation process. A qualitative synthesis and critical review of the literature was conducted in accordance with PRISMA‐ScR guidelines. The review protocol was registered with the Open Science Framework (osf.io/zfcuk).

**Results:**

After screening 219 citations, six studies from three independent research groups met the inclusion criteria. Sample sizes ranged from 40 to 192 donors. Five studies employed binary classification models using a 30% steatosis threshold, while one study reported a graded approach. Reported accuracies ranged from 0.81 to 0.92. Common challenges included small and imbalanced datasets with a dependence on supplementary donor data, such as blood tests and radiological findings. None of the studies conducted external validation.

**Discussion:**

Current evidence is drawn from a small and methodologically heterogeneous literature. Publications from several independent groups nevertheless highlight growing interest in developing these tools. Future work should prioritize larger studies with robust external validation to strengthen their credibility and build trust in their clinical use.

**Abbreviations**
AI: artificial intelligence
BMI: body mass index
DBD: donation after brainstem death
DCD: donation after cardiac death
ICU: intensive care unit
NORIS: National Organ Retrieval Imaging System
PRIMSA‐ScR: Preferred Reporting Items for Systematic Reviews and Meta‐Analyses extension for Scoping Reviews
SHAP: SHapley Additive exPlanations
TRIPOD+AI: Transparent Reporting of a multivariable prediction model for Individual Prognosis Or Diagnosis with Artificial Intelligence

## 1 Introduction

Maximizing organ utilization is a pressing challenge for the organ transplantation community [1, 2, 3]. The expanding donor demographics, characterized by an increase in Donations following Cardiac Death (DCD) and an aging population with a higher prevalence of comorbidities, have made organ utilization decisions more challenging [4, 5, 6]. In liver transplantation, hepatic steatosis is a critical factor that has been shown to predispose to microcirculatory impairment and more severe ischemia reperfusion injury, leading to poorer recipient outcomes [7, 8, 9]. Given the urgency of transplantation, clinicians frequently rely on visual assessments of steatosis to make utilization decisions, especially in settings where rapid biopsies are not available [10, 11].

Photography‐based remote assessment of donor livers has been common for many years, with Reddy et al. first reporting its use in 2008 through the National Organ Retrieval Imaging System (NORIS) [12]. In their pilot study, they showed that real‐time photo uploads enabled remote identification of grafts with significant steatosis with reliability similar to on‐site assessments. Despite improvements in image capture and standardized photography protocols [13, 14], this method of assessment remains highly dependent on surgical experience and is susceptible to inter‐rater and inter‐center variability, which can lead to sub‐optimal organ non‐utilization and extended waiting times [10, 15, 16].

The first reported attempt to apply computer vision and overcome the subjectivity of human liver assessments was reported by Thomson et al. in 2016, describing an automated image analysis system that calibrated and quantified liver quality from digital photographs taken at the time of organ donation [17]. Since then, advances in computer vision and artificial intelligence (AI) have further expanded the potential to standardize these assessments, making them more objective, reproducible, and reliable. Although there are promising initial studies from groups in the United Kingdom [18, 19], France [20, 21, 22], and Spain [23, 24], no comprehensive review has yet analyzed the current evidence on AI‐enabled visual assessment techniques for organ quality assessment. Given the emerging and diverse nature of this literature, a scoping review is the method of choice to map current research trends, understand the breadth of evidence, and highlight potential methodological gaps [25].

This scoping review aims to compile existing evidence on the use of AI for the photographic assessment of organ quality in liver transplantation. By comparing different approaches and their reported outcomes, the review will identify their strengths and limitations and outline priority areas for further research and development needed for clinical translation. This review also aims to equip researchers, clinicians, and policy makers with the necessary insights to consider and evaluate these emerging technologies.

## 2 Methods

A scoping review protocol was developed a priori following the Preferred Reporting Items for Systematic Reviews and Meta‐Analyses extension for Scoping Reviews (PRISMA‐ScR) [25]. The final protocol was registered prospectively with the Open Science Framework, (Protocol URL: https://osf.io/zfcuk) [26].

### 2.1 Study Selection and Search Strategy

Studies were eligible if they focused on the use of AI‐enabled computer vision techniques for assessing liver organ quality from photographs of livers taken during donation. Computer vision techniques were defined as algorithms that use the pixel data from digital liver photographs as their input and automatically extract visual features to train machine learning or deep learning models for classification, regression, or segmentation related to organ quality. Papers had to be published in English and report primary data from quantitative, qualitative, or mixed‐method studies. Studies were excluded if they did not address organ quality assessment using AI, were not peer‐reviewed, or fell outside the defined scope (e.g., editorials, commentaries).

A search was conducted across MEDLINE, EMBASE, and Scopus, covering literature from inception to March 27th, 2025. The search strategies were iteratively developed and refined through pilot searches to ensure comprehensive coverage of relevant studies (Table S1). In addition, we performed forward and backward citation searches of the included articles to identify any additional studies for inclusion. The final search results were exported for deduplication and the remaining records underwent title, abstract, and full‐text screening as outlined in our protocol [26].

### 2.2 Data Extraction

Two reviewers (G.K. and S.T.) independently completed title and abstract screening, reviewed full‐text articles and conducted data extraction. Any disagreements at any stage of the process were resolved through discussion and with the involvement of a third reviewer (C.W.) where necessary. For each included study, the following variables were extracted: first author, year of publication, title, country, sample size, type of computer vision task (e.g., binary classification, regression), metric of organ quality (e.g., steatosis on biopsy, surgeon visual assessment), any additional input variables used alongside imaging (e.g., donor demographics, laboratory values, imaging findings), timepoint and device used for image capture, method of image segmentation, and whether the dataset was balanced with respect to key features.

### 2.3 Data Synthesis and Analysis

A qualitative, descriptive synthesis consistent with PRISMA‐ScR guidance for scoping reviews was undertaken [25]. Study characteristics, AI methods, and performance metrics were charted in tabular form and then summarized narratively to compare approaches and identify common limitations or gaps. Critical appraisal of individual studies was conducted using the Transparent Reporting of a multivariable prediction model for Individual Prognosis Or Diagnosis with Artificial Intelligence (TRIPOD+AI) checklist [27]. The PRISMA flow diagram was constructed using the PRISMA Flow Diagram R Shinyapp [28].

## 3 Results

### 3.1 Study Selection and Characteristics

**FIGURE 1:**
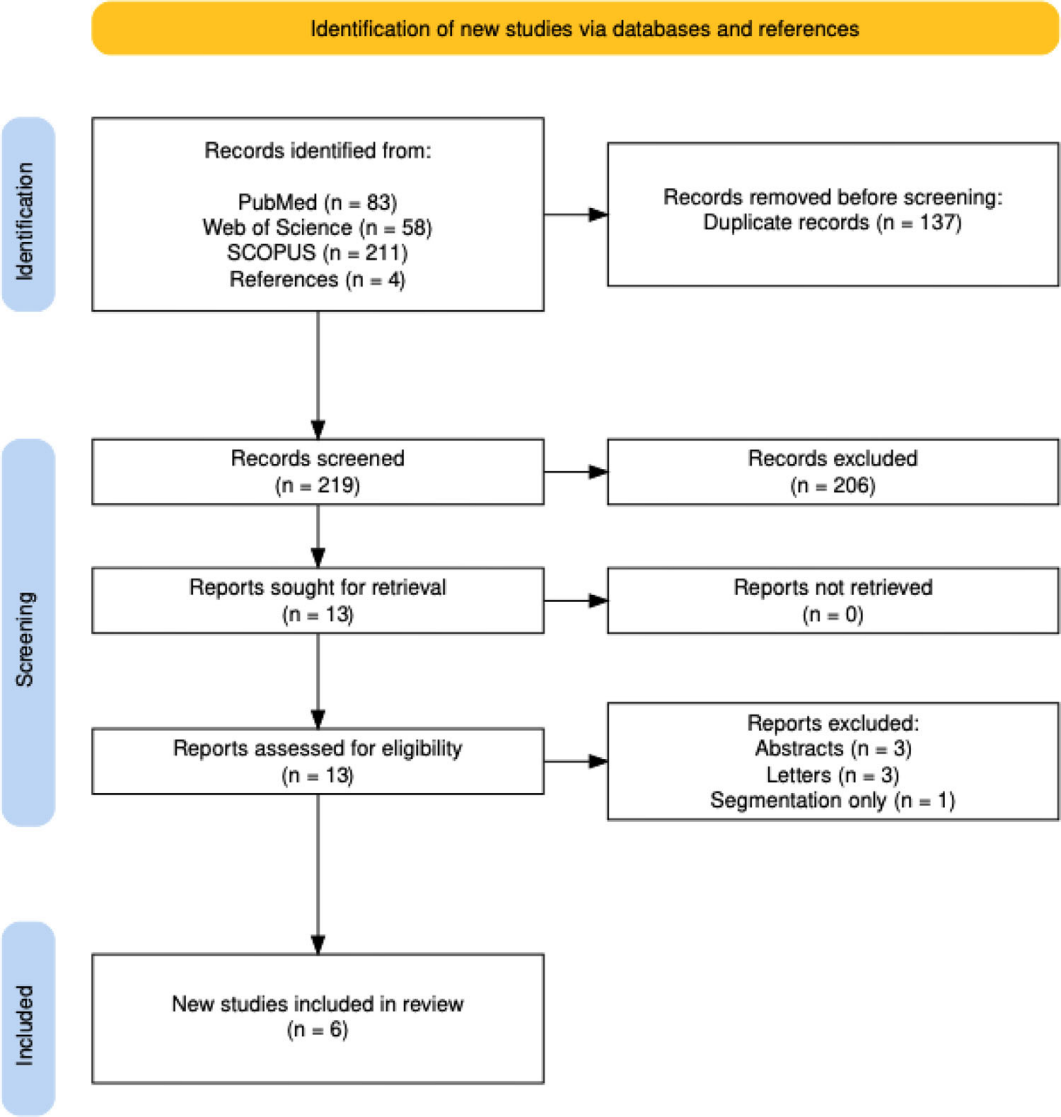
PRISMA flow diagram of study selection, inclusion, and exclusion.

A total of 219 unique citations were identified, with 206 excluded based on title and abstract screening. Of the 13 full texts reviewed, seven were excluded for reasons outlined in Figure 1. Six studies from three separate research groups across France (*n* = 3) [20, 21, 29], Spain (*n* = 2) [23, 24], and the United Kingdom (*n* = 1) [18] met the inclusion criteria for the review. A complete summary of the methodology and results for all included studies is outlined in Table 1.

**TABLE 1:** Summary of included studies, ordered by research groups.

Authors (Year)	Country	*n*	Computer vision task	Metric for assessing organ quality	Additional input variables	Performance	Image capture (time & device)	Image segmentation	Other
Moccia et al. (2018) [20]	France	40	Binary classification (Transplanted vs. not transplanted)	% macrovesicular HS on wedge biopsy & liver transplantability	Donor blood tests (not specified)	Sensitivity 0.95 Specificity 0.81 Accuracy 0.88	Liver in situ before aortic cross clamp with smartphone	Manual	‐ Balanced sample (*n* = 20 discarded livers with HS ≥60%, *n* = 20 transplanted livers with HS ≤20%) ‐ Accuracy reduced by 15% when excluding donor blood data (0.73 vs. 0.88)
Cesaretti et al. (2020) [21]	France	56	Binary classification (HS of <30% vs. ≥30%)	% macrovesicular HS on biopsy	Donor age, weight, height, GGT, ALT, AST, bilirubin, CT‐derived liver/spleen attenuation ratio	Accuracy 0.89 Sensitivity 0.97 for non steatotic livers Sensitivity 0.93 for steatotic livers	Liver in situ before aortic cross clamp with smartphone	Automatic	‐ Balanced sample (equal numbers of transplanted and not transplanted livers) ‐ 61 livers excluded from analysis to maintain balanced training and validation sets ‐ Performance of the model without additional donor data was not reported
Amer et al. (2021) [29]	France	154	Multiclass classification (HS of <30% vs. 30%–50% vs. >50%)	% macrovesicular HS on biopsy	None	Sensitivity 0.47 Specificity 0.74 Accuracy 0.81	Liver in situ before aortic cross clamp with smartphone	Manual	‐ Imbalanced dataset (*n* = 125 (81%) <30%, *n* = 14 (9%) 30%–50%, *n* = 17 (10%) >50% steatosis)
Ugail et at. (2022) [18]	United Kingdom	120	Binary classification (Transplantable vs. not transplantable)	Expert surgeon opinion of liver steatosis and transplantability	None	Accuracy 0.92 AUC 0.93	Backbench after cold flush with point‐and‐shoot camera	Automatic	‐ Balanced dataset (52% transplantable, 48% not transplantable)
Gómez‐Gavara et al. (2024) [24] and Piella et al. (2024) [23]	Spain	192	Binary classification (HS of <15% vs. ≥15% & <30% vs. ≥30%)	% macrovesicular HS on biopsy	Donor age, gender, BMI, AST, ALT, bilirubin, GGT, ultrasound‐assessed steatosis, ICU length of stay, and cause of death	15% threshold Accuracy 0.85 AUC 0.82 30% threshold Accuracy 0.85 AUC 0.74	Liver in situ before aortic cross clamp & backbench after cold flush with smartphone	Automatic	‐ Imbalanced dataset (*n* = 178 (92.7%) <30%, *n* = 14 (7.3%) >30% steatosis) ‐ Requires gray cards for white balancing ‐ SHAP analysis identified ultrasound‐assessed steatosis as the primary factor influencing predictions

Abbreviations: ALT, alanine aminotransferase; AST, aspartate aminotransferase; AUC, area under the curve; BMI, body mass index; CT, computed tomography; GGT, gamma‐glutamyl transferase; HS, hepatosteatosis; ICU, intensive care unit.

### 3.2 Photographic Assessment Approaches

The French research group used smartphone photographs of livers in situ before aortic cross‐clamping of Donors after Brainstem Death (DBD). Organ quality was determined by either liver utilization decisions [20] or the percentage of macrovesicular steatosis on wedge biopsy analysis [21, 29]. Sample sizes ranged from 40 to 157 donated livers. In the first two publications [20, 21], data balance was achieved by excluding patients from the majority group to ensure equal representation in both groups. In contrast, the last study [29] did not apply this method, with the majority group making up 81% of the cohort.

Their models used machine learning classifiers that processed color and texture features extracted from the photographs (full details in Table S2). The first study [20] employed a binary classification model based on liver utilization, the second [21] used binary classification with a 30% macrovesicular steatosis threshold, and the third study [29] implemented a multiclass model categorizing macrovesicular steatosis into three groups (<30%, 30%–50%, and >50%). The reported accuracies of the models ranged from 0.81 to 0.89. Of note, the first two studies [20, 21] enhanced their models by including additional donor data. The first [20] integrated donor blood tests, while the second [21] incorporated donor demographics, blood tests, and CT‐derived liver/spleen attenuation ratios, which resulted in improved model performance.

The two Spanish studies [23, 24] used both in situ photographs before aortic cross‐clamping and backbench photographs after cold preservation fluid flush, all captured with smartphones. Organ quality was assessed based on the percentage of macrovesicular steatosis determined by wedge biopsy analysis. The studies were based on the same cohort of 192 donated livers, 178 (93%) of which were classified in the <30% steatosis group.

The Spanish studies employed random forests and support vector machines to process color and texture features extracted from the photographs (full details in Table S2). Binary classification models were developed with threshold at 15% and 30% macrovesicular steatosis. The reported accuracy of the models was 0.85. Similar to the French group, they also incorporated additional donor data to enhance model performance, including donor age, gender, BMI, AST, ALT, bilirubin, GGT, ultrasound‐assessed steatosis, ICU length of stay, and cause of death. Notably, the Spanish group was the only one to use SHAP (SHapley Additive exPlanations) analysis—a machine learning interpretability technique [30]—to identify the most influential input variables in model predictions. Although both reports used the same model, the two SHAP analyses included in the publications differ. One analysis [23] included only BMI, donor age, and blood tests, while the other [24] also included the remaining donor data. In the latter SHAP analysis, donor gender and ultrasound‐assessed steatosis were identified as more influential than any of the variables in the former analysis. The photographic color and texture features were missing from both SHAP plots, making it impossible to determine how much the photographs contributed to the model's output.

The British research group [18] used photographs of livers taken on the backbench after a cold flush, captured with a point‐and‐shoot camera. Organ quality was determined based on expert surgeon visual assessments of macroscopic hepatic steatosis and whether the liver was considered transplantable or not. The study was conducted on a cohort of 120 livers, with a balanced split between those deemed transplantable and those not transplantable.

The computer vision approach by the British group involved comparing the performance of multiple supervised deep learning algorithms using patch segments extracted from the photographs (full details in Table S2). Binary classification models were developed to distinguish transplantable from non‐transplantable livers. A range of performance metrics were reported, with the highest accuracy reaching 0.92. The British group was the only one that did not incorporate additional donor data during model development.

### 3.3 Quality Assessment of Included Studies

The quality of the evidence was evaluated using the TRIPOD+AI checklist, with the full results provided in Table 2. Overall, the studies demonstrated similar strengths and weaknesses. Key limitations across the current literature include small sample sizes and a lack of external validation. Only one study reported 95% confidence intervals [23], and none of the studies included any information on the involvement of public and patient representatives in developing or evaluating these models.

**TABLE 2:** Quality assessment of the included studies using the TRIPOD+AI checklist for the reporting of prediction model studies.

Section	Item	Moccia et al. (2018) [20]	Cesaretti et al. (2020) [21]	Amer et al. (2021) [29]	Ugail et at. (2022) [18]	Gómez‐Gavara et al. (2024) [24]	Piella et al. (2024) [23]
Title	Title	+	**++**	+	**++**	+	**++**
Abstract	Abstract	+	+	+	+	+	+
Introduction	Background	**++**	**++**	**++**	**++**	**++**	**++**
	Objectives	**++**	**++**	**++**	**++**	**++**	**++**
Methods	Data	**++**	**++**	**++**	**++**	**++**	**++**
	Participants	+	**++**	+	**++**	**++**	**++**
	Data preparation	—	+	**++**	**++**	**++**	**++**
	Outcome	—	+	**++**	**++**	**++**	**++**
	Predictors	+	**++**	**++**	**++**	**++**	**++**
	Sample size	—	—	—	—	—	—
	Missing data	—	—	—	—	—	—
	Analytical methods	**++**	**++**	**++**	**++**	**++**	**++**
	Class imbalance	**++**	**++**	**++**	**++**	+	+
	Fairness	—	—	—	—	—	—
	Model output	**++**	**++**	**++**	**++**	**++**	**++**
	Training vs. evaluation	—	—	—	—	—	—
	Ethical approval	—	—	—	**++**	**++**	**++**
Open science	Funding	—	—	—	**++**	**++**	**++**
	Conflicts of interest	+	—	—	**++**	**++**	**++**
	Protocol	—	—	—	—	+	**++**
	Registration	—	—	—	—	**++**	**++**
	Data sharing	—	—	—	—	—	**++**
	Code sharing	—	—	—	—	—	—
Patient and public involvement	Patient and public involvement	—	—	—	—	—	—
Results	Participants	**++**	**++**	+	—	**++**	**++**
	Model development	**++**	**++**	**++**	**++**	**++**	**++**
	Model specification	**++**	**++**	**++**	**++**	**++**	**++**
	Model performance	+	+	+	+	+	**++**
Discussion	Interpretation	**++**	**++**	+	**++**	**++**	**++**
	Limitations	**++**	**++**	+	**++**	**++**	**++**
	Usability of model in context of current care	**++**	**++**	+	+	**++**	**++**

*Note:* **++,** complete adherence; +, partial adherence; —, not reported.

## 4 Discussion

There are currently six published studies from three independent groups that have examined AI‐enabled tools for the photographic evaluation of donated livers for transplantation. These studies provide early evidence that computer vision models can extract clinically meaningful visual features from liver photographs, whether captured in situ or on the backbench, to support organ assessment and utilization decisions. They also reveal heterogeneity in computer vision methodologies, the inclusion of supplementary input variables, and variability in the definitions and metrics used to assess liver quality.

Despite these promising initial results, several challenges must be addressed before these AI tools can translate into clinical practice. A key limitation is that most models use binary classification at a 30% steatosis threshold [18, 21, 23, 24]. This is a threshold of assessment already within the capability of experienced surgeons. In a cohort of 196 livers, Yersiz et al. reported that surgeon visual assessment at this threshold achieved 86.2% accuracy compared with histopathology [31]. To meaningfully expand safe organ utilization, AI tools need to improve discrimination within the >30% range, where decision‐making is more complex [6]. To date, only the French group has reported on a three‐class model that addresses this need [29].

Another limitation is the incorporation of additional donor data that may cause the models to rely more on these variables than on the photographs themselves. For example, ultrasound‐assessed steatosis was included as an input and appeared as the second most influential predictor after donor gender in the SHAP analysis by Gómez‐Gavara et al. [24]. Curiously, ultrasound‐assessed steatosis was absent from the SHAP analysis in the Piella et al. publication of the same model [23]. Moreover, neither analysis displayed the photographic color and texture features, making it impossible to judge how much the images contributed to the predictions. Although adding donor information can improve apparent performance, mandating variables such as CT or ultrasound limits generalizability and blurs the role of photographic input. If the stated aim is to develop tools that assess organ quality from routine photographs at donation, future work should clearly quantify the contribution of imaging alongside additional data. Otherwise, there is a risk that assessments will depend on these additional variables, leaving the photographic component effectively redundant.

The relatively small datasets used in these studies also present a challenge. Machine learning models typically require large amounts of data to improve performance. While data augmentation can help, extensive augmentation of a small dataset risks overfitting, may offer limited new variability, and can inadvertently reinforce biases present in the original datasets. Dataset imbalance further complicates model training and performance interpretation. Since most retrieved livers fall in the <30% steatosis category, a degree of data imbalance is inevitable. However, this imbalance can lead to the “accuracy paradox” where a model achieves high overall accuracy by consistently predicting the majority class while always failing to classify the minority class. We note that all of the reviewed publications recognized and addressed this challenge through various means.

A further concern is whether smartphone cameras capture sufficient detail compared to dedicated imaging systems. Evidence from surgical settings suggests that modern smartphones, aided by computational photography and automatic enhancement, are noninferior or even superior to dedicated cameras [13, 32]. This should reassure clinicians concerned about the development of these models using mobile device photography.

An additional point of discussion is that every AI developed so far fundamentally relies on macroscopic visual cues. Although visual assessment is not the gold standard, it remains central to time‐critical transplant decision‐making. In the United Kingdom, it remains the de facto standard approach for donated liver steatosis assessment [11]. Even in the United States, where pre‐donation liver biopsies are common, a recent national review of utilization spanning 2010–2021 found that 15.7% of livers were not recovered based on intraoperative visual evaluations compared with 6.0% due to biopsy findings [3]. Histological evaluation, while considered more objective, has its own limitations [33, 34]. Biopsy analysis services are not universally available around the clock; out‐of‐hours biopsy assessments are often conducted by non‐specialists, consensus on how to define and quantify steatosis remains limited [35, 36], and core biopsies risk sampling error [11, 37]. These issues weaken biopsy as a gold standard and support the case for exploring alternative approaches.

In the time‐critical transplant setting, AI tools that analyze routine liver photographs could offer a rapid, standardized, and non‐invasive method to estimate steatosis and organ quality. To earn the transplant community's trust and ensure adoption, they must be validated beyond expert grading and biopsy‐based steatosis estimates. Future work should also explore whether AI‑generated steatosis scores reliably predict post‑transplant outcomes and influence patterns of organ acceptance. Outcome‐driven validation is essential if photograph‐based AI tools are to move beyond proof‐of‐concept and meaningfully improve organ utilization.

The evolving landscape of liver transplantation is also being shaped by machine perfusion and advanced functional assessment techniques. Although distinct from AI photographic analysis, these technologies may have complementary roles. Given the cost and resource implications of machine perfusion, not all donated livers undergo functional assessment. AI‐driven visual assessment could help triage livers for immediate transplantation versus further evaluation, and in future may support real‐time assessment during perfusion.

This review is limited by the small and concentrated evidence base. Only six studies from three groups were eligible, reflecting an emerging field in which progress is constrained by the need for expert‐labeled datasets, linkage to histology or outcomes, strict data protection requirements, and skepticism about the value of macroscopic visual assessments. Finally, because of the small number and heterogeneity of studies, our findings should be interpreted as a descriptive overview rather than definitive estimates of model performance.

Transforming these AI tools from research prototypes into clinically useful decision aids will ultimately depend on whether clinicians, patients, and healthcare systems trust them. That trust will require external validation, participatory design processes that align technical development with clinical needs and patient expectations, and development that anticipates regulatory approval and builds in mechanisms for ongoing monitoring and oversight. This combination is essential if these models are to be viewed as ethically acceptable and implementable in transplant workflows.

In conclusion, our scoping review confirms that AI‐enabled photographic assessment tools have the potential to standardize the visual assessment of donor liver steatosis and reduce the subjective variability present in current practices. However, challenges such as binary classification models, dependence on supplementary donor data and small, imbalanced datasets lacking external validation, highlight the need for further development. Future development should progress beyond binary classification and incorporate robust external validation to improve the utility, credibility, and trust of these innovative techniques.

## Author Contributions

Concept and design: Georgios Kourounis, Colin Wilson, and Neil S. Sheerin. Data cleaning and synthesis: Georgios Kourounis and Samuel J. Tingle. Data interpretation: All authors. Drafting of the article: Georgios Kourounis. Critical revision and approval of the article: All authors

## Conflicts of Interest

The authors (G.K., A.E., B.T., H.U., and C.W.) have been supported by an NIHR Invention for Innovation grant (NIHR204169) to develop an AI‐enabled photographic organ quality assessment tool in transplantation. The grant had no role in the study design, data collection, analysis, interpretation, or decision to publish.

## Supporting information




**Supplementary Table 1**. Search strategies
**Supplementary Table 2**. Specific computer vision methodologies employed across the included studies.

## Data Availability

Data sharing is not applicable to this article as no datasets were generated or analyzed during the current study.
