# Supplementary material for: A Scoping Review of the Photographic Assessment of Donor Liver Steatosis in Transplantation Using Artificial Intelligence
Source: Clin Transplant. 2026 Jan 31;40(2):e70433. doi: 10.1111/ctr.70433 (PMC12859739; doi:10.1111/ctr.70433)
Supplement: Supplementary file 1 — Supplementary Table 1. Search strategies Supplementary Table 2. Specific computer vision methodologies employed across the included studies. [file CTR-40-e70433-s001.docx]

**A scoping review of photographic assessment of donor liver steatosis in transplantation using artificial intelligence – Supplementary Material**

Georgios Kourounis^1,2,3^, Samuel J Tingle^1,2,3^, Ali Elmahmudi^4^, Brian Thomson^4^, Robin Nandi^5^, Emily Thompson^1,2,3^, Barney Stephenson^3^, James Hunter^6^, Hassan Ugail^4^, Neil S Sheerin^1,2,3^, Colin Wilson^1,2,3^

**Supplementary Table 1.** Search strategies

**Supplementary Table 2**. Specific computer vision methodologies employed across the included studies.

**Supplementary Table 1:** Search Strategies

| **Database** | **Search Strategy** |
| --- | --- |
| **PubMed**  **and**  **Web of Science** | ((liver OR graft OR donor) AND  (transplant* OR donation OR procur*) AND  (photo* OR image* OR smartphone OR colo*) AND  (steato* OR "fatty liver" OR macrosteatosis) AND  (AI OR "artificial intelligence" OR "deep learning" OR "machine learning" OR "computer-assisted" OR "computer vision")) |
| **SCOPUS** | TITLE-ABS-KEY(  (  (liver OR graft OR donor)  AND (transplant* OR donation OR procur*)  AND (photo* OR image* OR smartphone OR colo*)  AND (steato* OR "fatty liver" OR macrosteatosis)  AND (ai OR "artificial intelligence" OR "deep learning" OR "machine learning" OR "computer-assisted" OR "computer vision")  )  ) |

**Supplementary Table 2**. Specific computer vision methodologies employed across the included studies.

| **Authors (Year)** | **Reported methodology** |
| --- | --- |
| Moccia et al.  (2018)^20^ | Texture analysis using histogram of local binary patterns (LBP), grey-level co-occurrence matrix (GLCM), and intensity features; classified with semi-supervised learning, in addition to blood test data (not specified). |
| Cesaretti et al.  (2020)^21^ | Automatic liver segmentation and classification using machine learning (SVM-SIL) with donor data; texture and colour analysis of smartphone images. Additional inputs: donor age, weight, height, blood tests (GGT, ALT, AST, bilirubin), and CT-derived liver/spleen attenuation ratio |
| Amer et al. (2021)^29^ | Colour and texture feature extraction in RGB, HSV, and YCbCr spaces using LBP, LPQ, and GLCM; classification using LASSO regression. No additional clinical inputs used |
| Ugail et at. (2022)^18^ | Transfer learning with pre-trained deep learning models (VGGFace, VGG16, ResNet50, DenseNet121, MobileNet) for feature extraction; classification using SVM, logistic regression, and decision trees. No additional input data used |
| Gómez-Gavara et al.  (2024)^24^  & Piella et al. (2024)^23^ | Colour and texture analysis of smartphone images using random forest and SVM classifiers; applied to image patches after colour calibration using grey cards. Additional inputs: age, gender, BMI, blood tests (AST, ALT, GGT, bilirubin), ultrasound-assessed steatosis, ICU stay, and cause of death |
